# ﻿A new species of *Metaurus* Stål, 1866 (Hemiptera, Fulgoromorpha, Dictyopharidae), supplemented with mitogenome data from China

**DOI:** 10.3897/zookeys.1215.128976

**Published:** 2024-10-11

**Authors:** Yan-Li Zheng, Thierry Bourgoin, Lin Yang, Xiang-Sheng Chen

**Affiliations:** 1 School of Geography and Resources, Guizhou Education University, Guiyang, Guizhou, China 550018, China; 2 The Provincial Key Laboratory for Agricultural Pest Management of Mountainous Region; Institute of Entomology, Guizhou University, Guiyang, Guizhou 550025, China; 3 Guizhou Provincial Key Laboratory of Geographic State Monitoring of Watershed, Guizhou Education University, Guiyang 550018, China; 4 Mountain Research Institute, Guizhou Education University, Guiyang, Guizhou, China 550018, China; 5 Institut Systématique, Evolution, Biodiversité (ISYEB), MNHN-CNRS-Sorbonne Université-EPHE-Univ. Antilles, Museum National d'Histoire Naturelle,75005, Paris, France

**Keywords:** Auchenorrhyncha, distribution, Fulgoroidea, identification key, morphology, planthoppers, taxonomy

## Abstract

A new planthopper species, *Metaurusmohanensis* Zheng & Chen, **sp. nov.**, is described and illustrated from Yunnan, China. A key to differentiate species within the genus *Metaurus* is provided. The geographical distribution of *Metaurus* species and mitochondrial genome data of the newly described species are also included.

## ﻿Introduction

With a global distribution of 748 species across 160 genera (Bourgoin 2024), the family Dictyopharidae Spinola, 1839 represents a moderately sized taxon within the Hemiptera: Fulgoromorpha. Like all planthoppers, Dictyopharidae are strictly phytophagous insects, known to feed on over 25 families. Their diet predominantly includes dicots, particularly Asterales (13%), Caryophyllales (9%), Fabales (9%), and Fagales (7%), as well as monocots such as Poales (13%) and Liliales (7%) (Bourgoin 2024). Several dictyopharid species are recognized as economically significant agricultural pests (Wilson and O’Brien 1987; Wilson et al. 1994).

Dictyopharidae is currently classified into 19 tribes, plus two fossil ones, divided between two subfamilies: Dictyopharinae Spinola, 1839 and Orgeriinae Fieber, 1872 (Song et al. 2018; Bourgoin 2024). The genus *Metaurus* Stål, 1866 was initially placed in the tribe Dictyopharini Spinola, 1838, but was transferred to Orthopagini Emeljanov, 1983 by Emeljanov (2011) and later revised by Song and Liang (2012). In this latter study, the authors described a new species, *M.ramusitis* Song & Liang, 2012 from Laos and China (Yunnan), in addition to the type species *M.reticulatus* Stål, 1866 known from Cambodia. They also identified an unnamed species from Thailand, which is definitively distinct but could not be formally described due to the absence of its abdomen.

We formally describe here a third species of *Metaurus* from Yunnan, China, *M.mohanensis* sp. nov. In addition to its morphological description, we also provide its complete mitogenome.

## ﻿Materials and methods

### ﻿Morphology

The specimens examined have been deposited in the Institute of Entomology, Guizhou University, Guiyang, China (GUGC). Dry specimens were used for the descriptions and illustrations. Genital segments of the specimens were macerated in a boiling solution of 10% NaOH, transferred to preparations of glycerin jelly, and examined under a Leica MZ12.5 stereomicroscope. Photographs of adult habitus were obtained using a Keyence VHX-1000 system. Illustrations were scanned with Canon Cano Scan LiDE 200 and imported into Adobe Photoshop CS6 for labeling and composition of figures.

The morphological terminology follows Yang and Yeh (1994) for the head and body, Bourgoin et al. (2015) for the forewing venation, and Bourgoin (1987, 1993) and Yang and Yeh (1994) for male and female genitalia, respectively. The usual standardized notation is used for the wing venation as follows: A1 first anal vein; bc, basal cell; CuA cubitus anterior; CuP cubitus posterior; MP: media posterior; R: radius; Sc: subcosta. The following abbreviations are used in the text for measurements: BL body length (from apex of cephalic process to tip of forewings); HL head length (from apex of cephalic process to base of eyes); HW head width (including eyes); FWL forewing length.

Biogeographical realms are named according to Holt et al. (2013).

### ﻿Molecular methods

Total genomic DNA was extracted from the muscle tissue from the hind legs of the holotype specimen using the Takara Genome DNA Extraction Kit (Sangon Biotech, Shanghai, China) according to the manufacturer’s instructions. The mitogenome was sequenced using a next-generation sequencing platform with Illumina Hiseq 2500 at OriGene (Beijing, China).

The quality of the raw sequences was evaluated using FastQC v.0.11.4 (www.bioinformatics.babraham.ac.uk/projects/fastqc). Putative mitogenome reads with an average quality value of < Q30 were removed before assembly. Then, the clean sequences were assembled using MitoZ v.2.4 software with default parameters and the mitogenome of *Orthopagussplendens* (Fulgoroidea: Dictyopharidae; GenBankNo. MW441850) as a reference (Zheng et al. 2021). The mitogenome was initially annotated using Galaxy Community 2022 with invertebrate genetic codes. PCGs were predicted by determining their open reading frames using the invertebrate mitochondrial genetic codon. rRNA genes and the AT-rich regions were determined by comparisons with the homologous sequences of other planthoppers from GenBank. Mitogenomic circular maps were depicted and annotated using Geneious R9. Strand asymmetry was calculated according to the following formulas: AT skew = (A-T)/(A+T) and GC skew = (G-C)/(G+C). The mitogenome sequences of the new species have been deposited in GenBank under the accession number PP863288.

## ﻿Taxonomy

**Subfamily Dictyopharinae Spinola, 1839**

**Tribe Orthopagini Emeljanov, 1983**

### ﻿Genus Metaurus

Taxon classificationAnimaliaHemipteraDictyopharidae

Stål, 1866

965D01B5-2E25-527F-8F67-1BBB1CDD892E

Metaurus Stål, 1866a: 151. Type species: Metaurusreticulatus Stål, 1866; by subsequent designation by Melichar (1912).
Metaurus Stål: Stål 1866b: 391; Atkinson 1886: 24; Melichar 1912: 46; Metcalf 1946: 38; Song and Liang 2012: 2564.

#### Diagnosis.

See Song and Liang (2012).

#### Distribution.

Oriental region (Cambodia; Laos; southwestern China; Thailand) (Song and Liang 2012).

### ﻿Key to the species of *Metaurus* Stål (modified from Song and Liang 2012)

**Table:** 

1	In lateral view, cephalic process in front of eyes distinctly longer than distance from curved point to posterior margin of eyes, with the ratio about 1.4: 1	***M.* indet. sp.**
–	In lateral view, cephalic process in front of eyes slightly shorter or as long as distance from curved point to posterior margin of eyes	**2**
2	In lateral view, cephalic process in front of eyes slightly shorter than distance from curved point to posterior margin of eyes, with the ratio about 0.7–0.8: 1. In dorsal view, segment X of males short and small, with ratio of the maximum length to width near base about 1.1: 1. In lateral view, apical ventral margins protruded ventrally into a small process. Aedeagus with ventral outer apical lobes curved anteriorly in ventral view	***M.ramusitis* Song & Liang**
–	In lateral view, cephalic process in front of eyes as long as distance from curved point to posterior margin of eyes. In dorsal view, segment X of males large and elongate, with ratio of the maximum length to width near base > 1.5. In lateral view, apical ventral margins protruded ventrally into a large rounded process. Aedeagus with ventral outer apical lobes directed posteriorly in ventral view	**3**
3	In dorsal view, segment X of males with ratio of the maximum length to width near base about 1.6: 1; its ventral margins irregularly incised at midlength in lateral view. Aedeagus with ventral outer apical lobes directed posteriorly in ventral view	***M.reticulatus* Stål**
–	In dorsal view, segment X of males with ratio of the maximum length to width near base about 2.3: 1; its ventral margins only slightly concave, not incised at midlength in lateral view. Aedeagus with dorsal apical lobes (Figs 11, 16) produced in a pair of long lobes, directed posteriorly in lateral view, and two lobes on it, base extends forward to form a leaf projection; ventral lobes (Figs 10, 17) with two pairs of lobes	***M.mohanensis* Zheng & Chen, sp. nov.**

### ﻿ Metaurus mohanensis

Taxon classificationAnimaliaHemipteraDictyopharidae

Zheng & Chen sp. nov.

CCD763D0-C980-5CA9-9F20-00F57A144462

https://zoobank.org/9761682E-75C4-449E-BF12-10C12713C931

#### Type material.

***Holotype*** • ♂, China: Yunnan, Xishuangbanna Mengla County, Mohan, 16 June 2019, Feng-E Li (GUGC, no. GUGC-20220811-Y13). ***Paratypes*** • 2♂♂, same collection site as the holotype but 22 June 2019, Yalin Yao (GUGC).

#### Diagnosis.

The new species is similar to *Metaurusramusitis* Song & Liang, but can be distinguished from the latter by the shape of the phallobase and segment X: dorsal apical lobes of the phallobase (Figs 11, 16) produced in a pair of long lobes, directed posteriorly in lateral view, and two lobes on it, base extends forward to form a leaf projection in dorsal view (not in *M.ramusitis*). Segment X in dorsal view is relatively narrow, with ratio of the maximum length to width near base about 2.3: 1 (Fig. 13); ventral margin is protruded ventrally into a large rounded process at base in lateral view and slightly concave, not incised medially (Figs 8, 12); in *M.ramusitis*: segment X is relatively broader in dorsal view, and with an irregular incision medially in lateral view.

#### Description.

Measurements (in mm). ♂, BL: 17.3–17.1 mm; HL: 3.6–3.7 mm; HW: 1.7–1.8 mm; FWL: 12–12.1 mm.

***Coloration*.** Body green. General colour greenish ochraceous; darker on apical part of cephalic process, on a longitudinal spot before eyes on genae, and on a small anterior spot on lower lateral carina behind eyes on pronotum. Rostrum blackish at extreme apex. Fore and hind wing membrane transparent, veins green, stigmal area green, anterior margin yellow. Legs green, apex of hind femora and base of hind tibiae black, tips of lateral spines on hind tibiae and tips of apical teeth on tarsomeres black.

**Figures 1–7.**
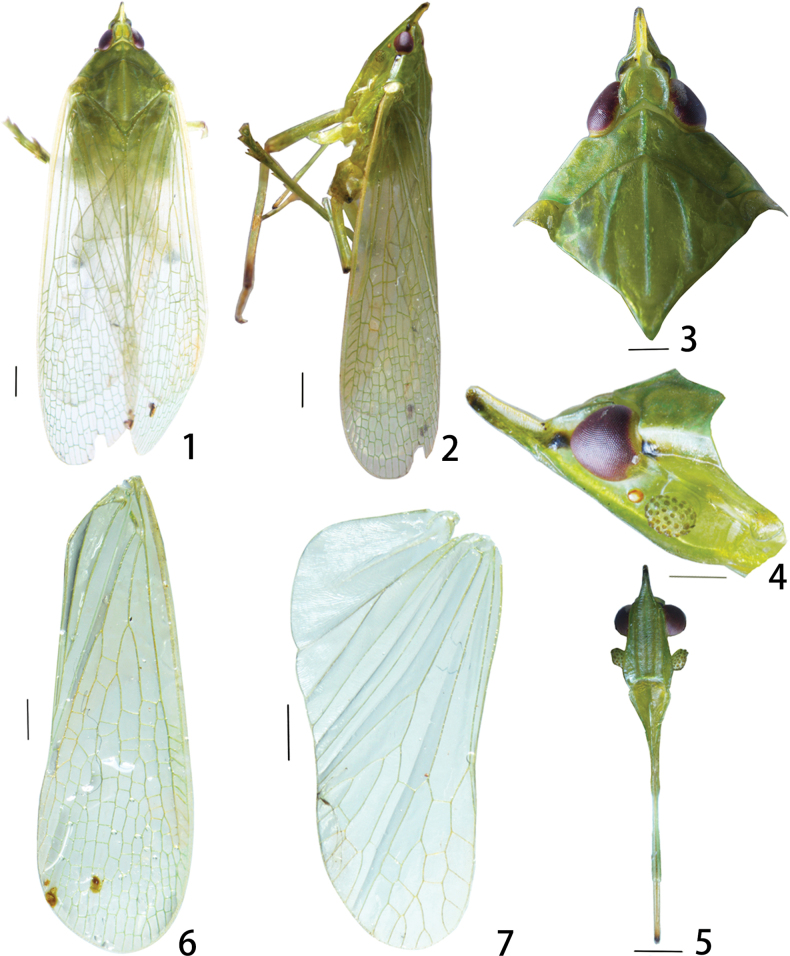
Metaurusmohanensis Zheng & Chen, sp. nov. **1** male, holotype, dorsal view **2** male, lateral view **3** male, head and thorax, dorsal view **4** male, frons and clypeus, ventral view **5** male, head and pronotum, lateral view **6** male, forewing **7** male, hind wing. Scale bars: 1 mm.

***Morphology*.** Head and thorax (Figs 1–5). Cephalic process in front of eyes strongly upturned (about 45°), as long as distance from curved point to posterior margin of eyes in lateral view. Head shorter than pronotum and mesonotum combined, its ratio of length: width about 1:0.45 . Vertex relatively narrow, median longitudinal carina distinct only at the base, and lateral carina deeply concave and strongly tapered in front of the compound eyes. Frons elongate, with median carina complete and elevated, 2.5 times as long as wide, anterior portion distinctly narrowed and protruded anteriorly and upwardly in ventral and lateral views; lateral carinae ridged, frons distinctly expanded outwards below antennae. Postclypeus and anteclypeus strongly convex at middle, with distinct median carina. Rostrum long, extending up to abdominal sternite VI. Pronotum distinctly shorter than mesonotum medially, ratio length about 0.4:1, narrow anteriorly, broad posteriorly. Mesonotum tricarinate, lateral carinae incurved anteriorly towards median carina.

***Forewings*** (Figs 1, 2, 6) hyaline, much longer than abdomen, nearly three times as long as broad; anterior margin distinctly expanded into a narrow, sclerotized costal area; costal cell without transverse veins, Sc+R forked apicad of junction of claval veins; MP first branching in MP_1+2_ and MP_3+4_ veins near basal one-third, anterior to first CuA branching, MP_1+2_ fork situated posterior to MP_3+4_ fork and then branching into a dozen of terminals; numerous transverse veins among Sc+R, MP and CuA on apical two-thirds; 22 apical cells; Pcu and A1 fused into a short Pcu+A1 vein at apical third of clavus; pterostigmal area not differentiated, elongate, with nine cells.

***Hindwings*** (Fig. 7) hyaline. CuA first branching into CuA_1_ and CuA_2_ near middle; MP and CuA_1_ branching to several accessory veins on apical two-thirds, with several transverse veins; 14 apical cells.

***Legs*** narrow and long, fore femora flattened and dilated, with a distinct spine near apex, hind tibiae with 7 lateral spines and 7 apical teeth, hind tarsomeres I with 10 and tarsomeres II with 11 apical teeth. Metatibiotarsal formula: 7-7/10:11

**Figures 8–11.**
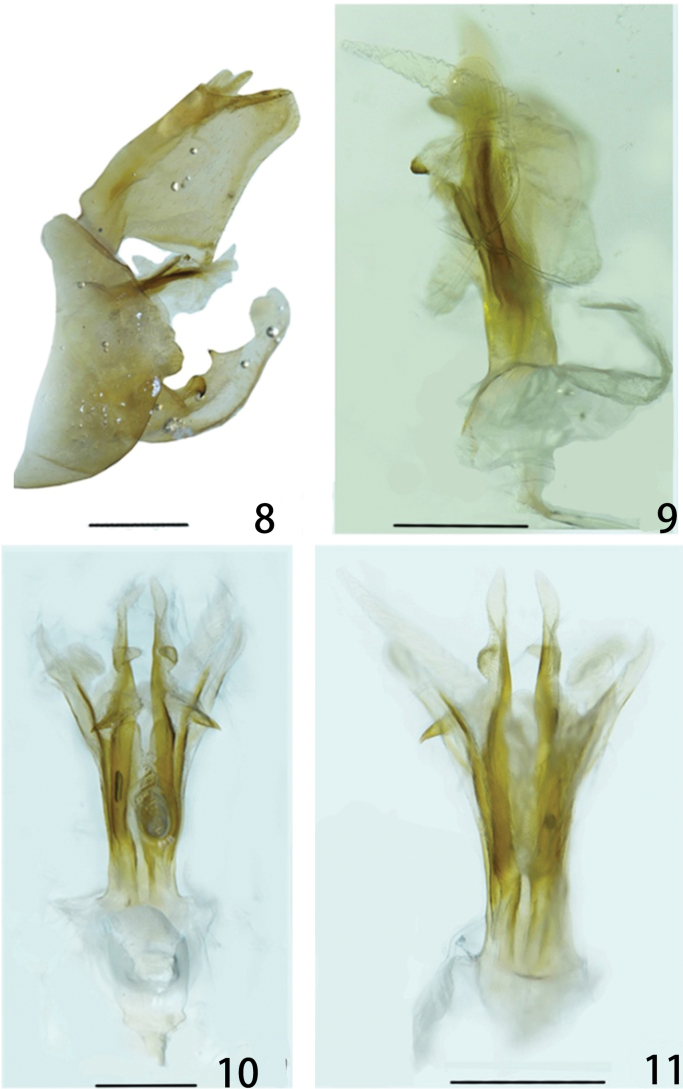
*Metaurusmohanensis* Zheng & Chen, sp. nov. **8** genitalia, lateral view **9** aedeagus, lateral view **10** aedeagus, ventral view **11** aedeagus, dorsal view. Scale bars: 1 mm (**8**); 0.5 mm (**9–11**).

**Figures 12–17.**
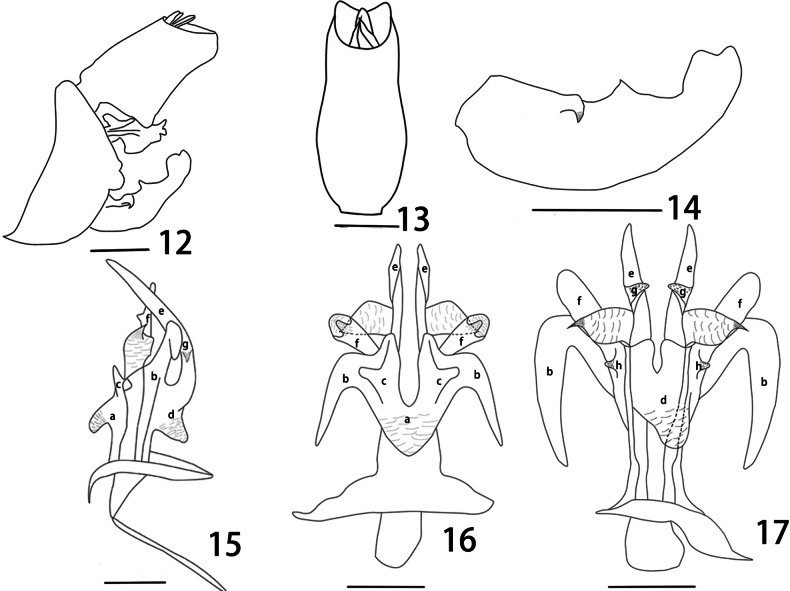
*Metaurusmohanensis* Zheng & Chen, sp. nov. **12** genitalia, lateral view **13** segment X, dorsal view **14** gonostyles, lateral view **15** aedeagus, lateral view **16** aedeagus, dorsal view **17** aedeagus, ventral view **a–h** 3 pairs of apical membranous lobes (same letters representing the same parts). Scale bars: 1 mm (**12**); 0.5 mm (**13–17**).

***Male genitalia*.** Pygofer large and broad, ventrally distinctly longer than dorsally (about 5: 1); posterior margin with two obtuse processes near middle in lateral view (Figs 8, 12), upper process slightly smaller than lower one. Segment X (anal tube) large and broad in dorsal view, with ratio of the maximum length to width near base about 2.3: 1 (Figs 8, 12, 13); ventral margins in basal third protruded ventrally into a large triangular process in lateral view. Gonostyli relatively small in lateral view, posterior margin with directed tooth-like dorsal process medially and a directed tooth-like ventral process submedially on outer upper edge; apical part elongate and rounded (Figs 8, 12, 14); dorsal process short, acute apically. Aedeagus (Figs 9–13) large and robust, dorsal and lateral parts and most of ventral part of phallobase sclerotized and pigmented, the remainder membranous; with 3 pairs of apical membranous lobes; dorsal apical lobes (Figs 15, 16) long, directed posteriorly in lateral view, each bearing two smaller projections (Figs 15c, 16c) on it, base extending forward to form a leaf-like projection (Figs 15a, 16a); ventral lobes (Figs 10, 15, 17) large and slender, with two pairs of projections (Figs 10, 15, 17): the inner ones very slender (Figs 15f, 17f), directed posteriorly, with additional two smaller membranous projections on their median part (Fig. 17h), and one large basal lobe (Figs 15d, 17d); the outer ones slender and elongate, directed posteriorly (Fig. 17e), with two small projections medially in ventral view (Figs 15g, 17g).

#### Etymology.

The species name “*mohanensis*” refers to the collecting site in the town of Mohan, in the Yunnan Province in southwestern China. Adjective.

##### ﻿Mitochondrial genome of the new species

**Figure 18.**
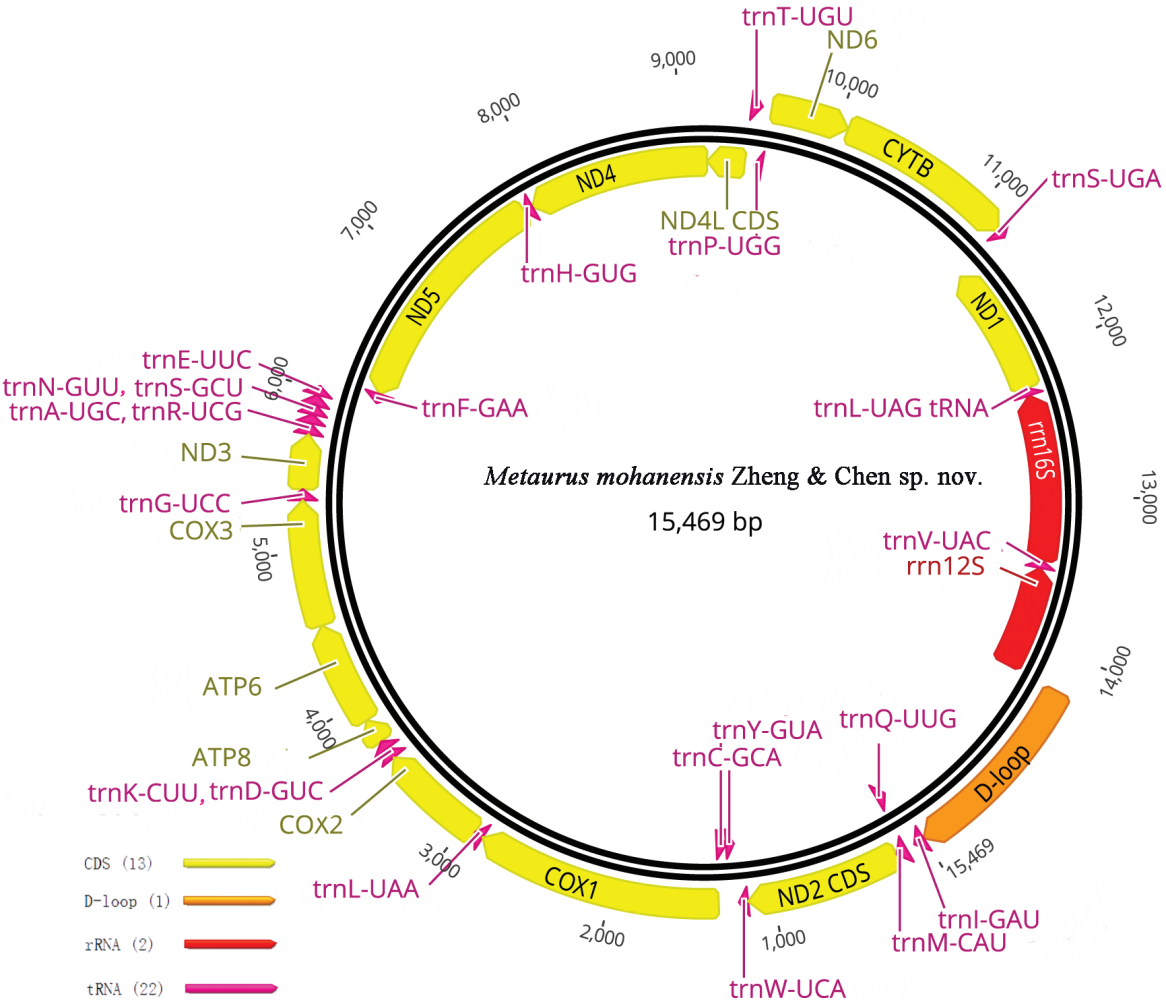
Circular map of the mitogenome of *Metaurusmohanensis* Zheng & Chen, sp. nov.

The complete mitogenome of *Metaurusmohanensis* Zheng & Chen, sp. nov. is 15,469 bp in length and consists of 13 protein-coding genes (PCGs), 2 ribosomal RNA genes (rRNAs), 22 transfer RNA genes (tRNAs) and one large non-coding region (D-loop: [A+T]-rich region) (Fig. 18). The D-loop part is 1233 bp, taking place between 12S rRNA and trnI. The overall base composition is A: 48%, T: 28.8%, C: 15%, and G: 8.2%. AT skew ((A-T)/(A+T)) and GC skew ((G-C)/(G+C)) are 0.25 and - 0.293, respectively. All 13 PCGs started with ATN, GTG (nad1, nad5) and ended with TAN or a single T (nad1, nad4, atp6 and nad5) residue. The length of 22 tRNA ranged from 57 bp (trnV) to 70 bp (trnK). Genes of 16S rRNA and 12S rRNA are 1, 201 bp and 732 bp, respectively.

The genomic data from this study are openly available in the National Center for Biotechnology Information (NCBI) at https://www.ncbi.nlm.nih.gov, accession number: PP863288. Associated BioProject, SRA, and BioSample accession numbers are PRJNA1114399, SUB14549433 and SAMN41484364 respectively (https://www.ncbi.nlm.nih.gov/bioproject/PRJNA1114399).

## ﻿Discussion and conclusions

**Figure 19.**
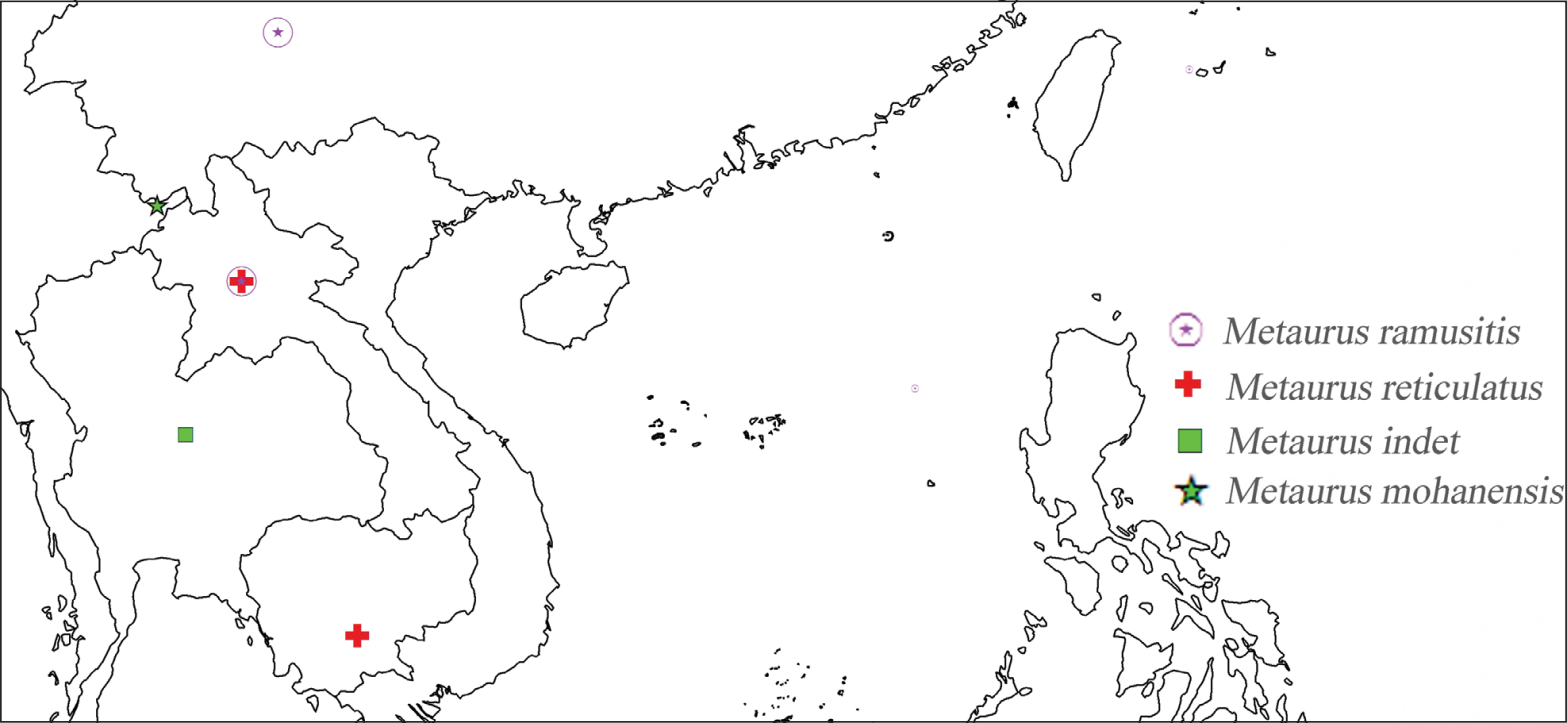
Geographical distribution of *Metaurus* species.

The Oriental genus *Metaurus* exhibits a continental distribution in southeastern Asia, being restricted to Thailand, Cambodia, Laos and southwestern China(Fig. 19). *Metaurusramusitis* Song & Liang, 2012 is known from Laos and only by one female from Yunnan, China. The new species in this study was also collected from Yunnan, China.

Species of *Metaurus* are externally quite similar to those of *Centromeria* Stål, 1870, a more diversified genus of Orthopagini with 14 species known to date (Song et al. 2016; Bourgoin 2024), but with a wider southeastern Asian distribution corresponding to the Oriental zoogeographic region as defined by Holt et al. (2013). *Metaurus* can be separated from *Centromeria* by the following characters: 1) Head in front of the eyes is strongly upturned, forming a slender, straight process, with the vertex’s median carina only weakly ridged at the base (versus a frontal process that is moderately to strongly curved upwards in *Centromeria*); the vertex in *Centromeria* has lateral carinae that are moderately or abruptly constricted, strongly upturned in front of the eyes, and then gradually convergent anteriorly, culminating in an acuminate apex; 2) MP vein first forks into MP_1+2_ and MP_3+4_ veins before CuA, close to the basal one-third of corium, which is branched and with numerous accessory veins on the apical two-thirds of the corium (while MP bifurcating into MP_1+2_ and MP_3+4_ near middle, and posterior to CuA in *Centromeria*); and 3) Hind tibiae in *Metaurus* have 7 apical teeth, compared to only 6 apical teeth in *Centromeria*.

The mitogenome of *Metaurusmohanensis* Zheng & Chen, sp. nov. differs from *Orthopagussplendens* (Fulgoroidea: Dictyopharidae: Orthopagini; GenBankNo. MW441850) (Zheng, Bourgoin et al. 2021) by the following characteristics: 1) full length 15469 (15346 in *O.splendens*); 2) overall base composition: A: 48%, T: 28.8%, C: 15%, and G: 8.2%. AT skew ((A-T)/(A+T)) and GC skew ((G-C)/(G+C)) 0.25 and - 0.293, respectively (versus A: 47.2%, T: 30.2%, C: 14.6%, and G: 8%. AT skew ((A-T)/(A+T)): 0.22 and GC skew ((G-C)/(G+C)): - 0.294 in *Orthopagussplendens*); and 3) In *M.mohanensis*, all 13 PCGs start with ATN or GTG (nad1, nad5) and end with TAN or a single T (nad1, nad4, atp6 and nad5) residue. The length of 22 tRNA ranges from 57 bp (trnV) to 70 bp (trnK). Genes of 16S rRNA and 12S rRNA are 1,201 bp and 732 bp, respectively. In *O.splendens*, all 13 PCGs start with ATN and end with TAN or a single T (nad1, nad5 and atp6) residue. The length of 22 tRNA ranges from 59 bp (trnS) to 70 bp (trnK). Genes of 16S rRNA and 12S rRNA are 1,177 bp and 729 bp, respectively.

## Supplementary Material

XML Treatment for Metaurus

XML Treatment for Metaurus mohanensis

## ﻿Additional information

### Author ORCIDs

Yan-Li Zheng https://orcid.org/0000-0002-6964-5988

Thierry Bourgoin https://orcid.org/0000-0001-9277-2478

### Data availability

All of the data that support the findings of this study are available in the main text.
